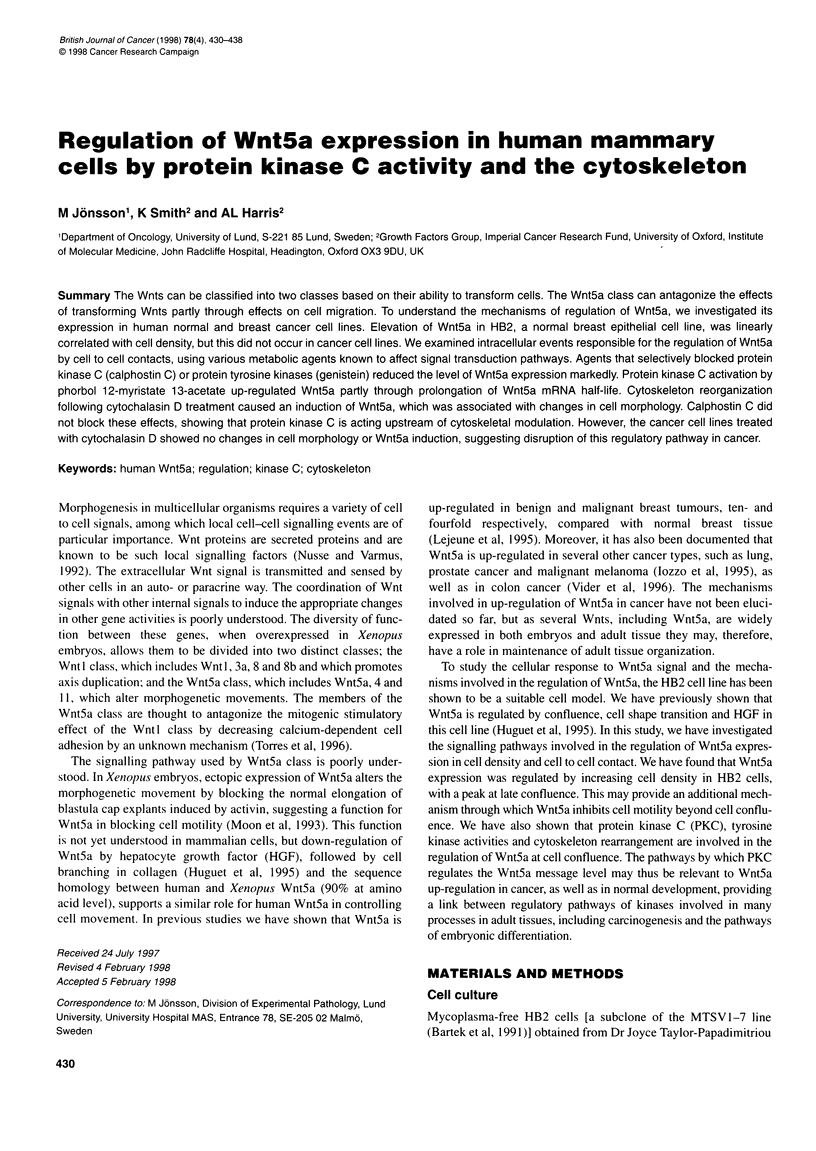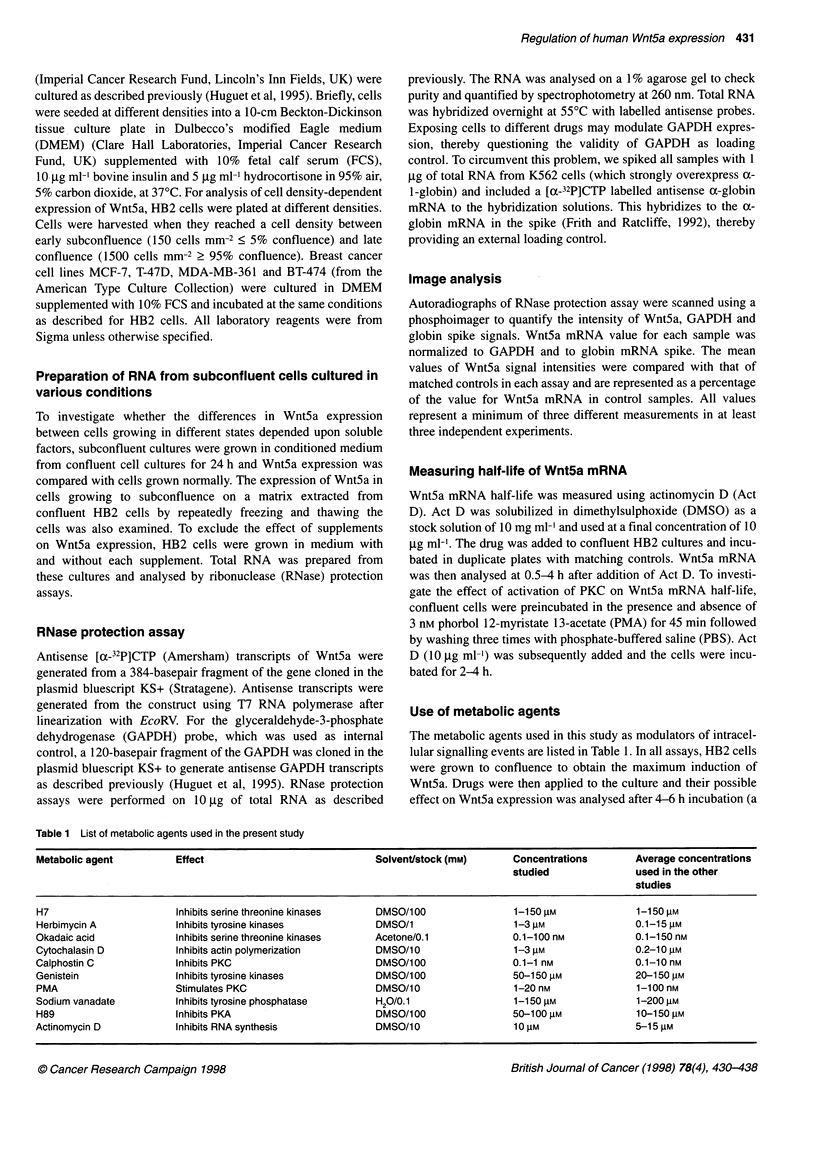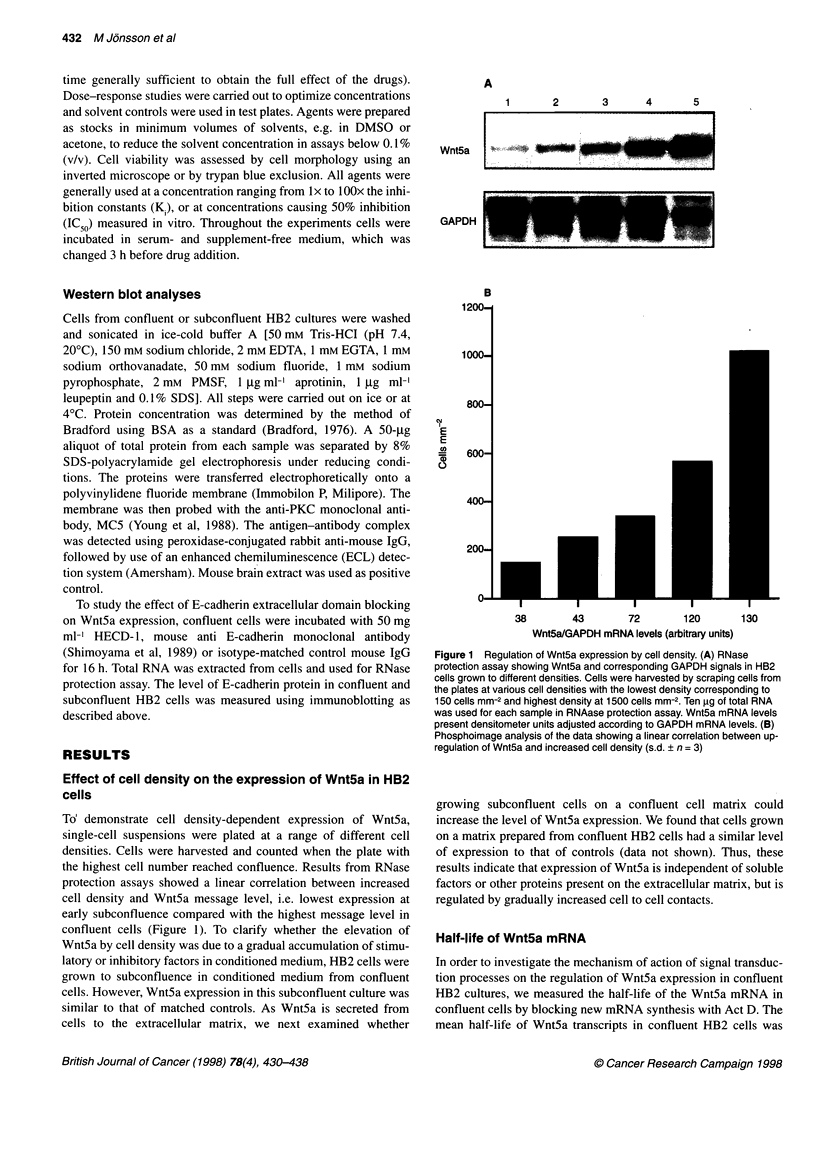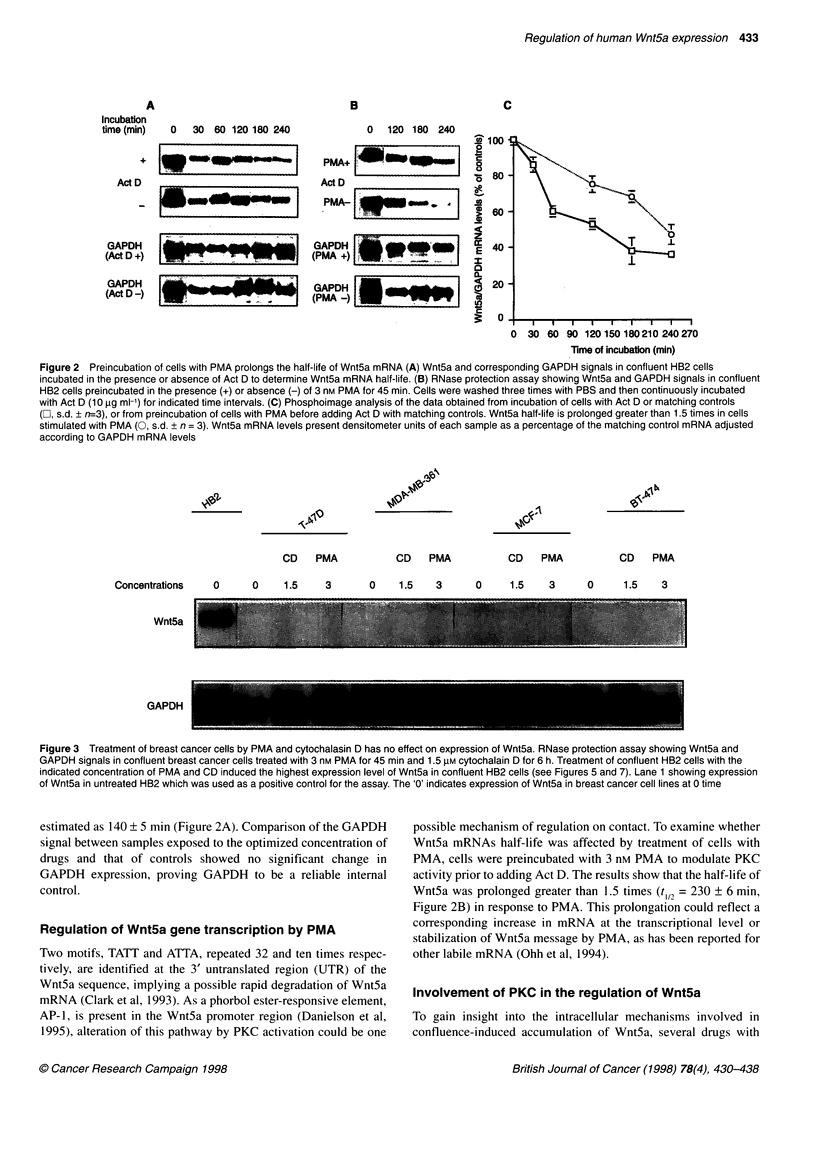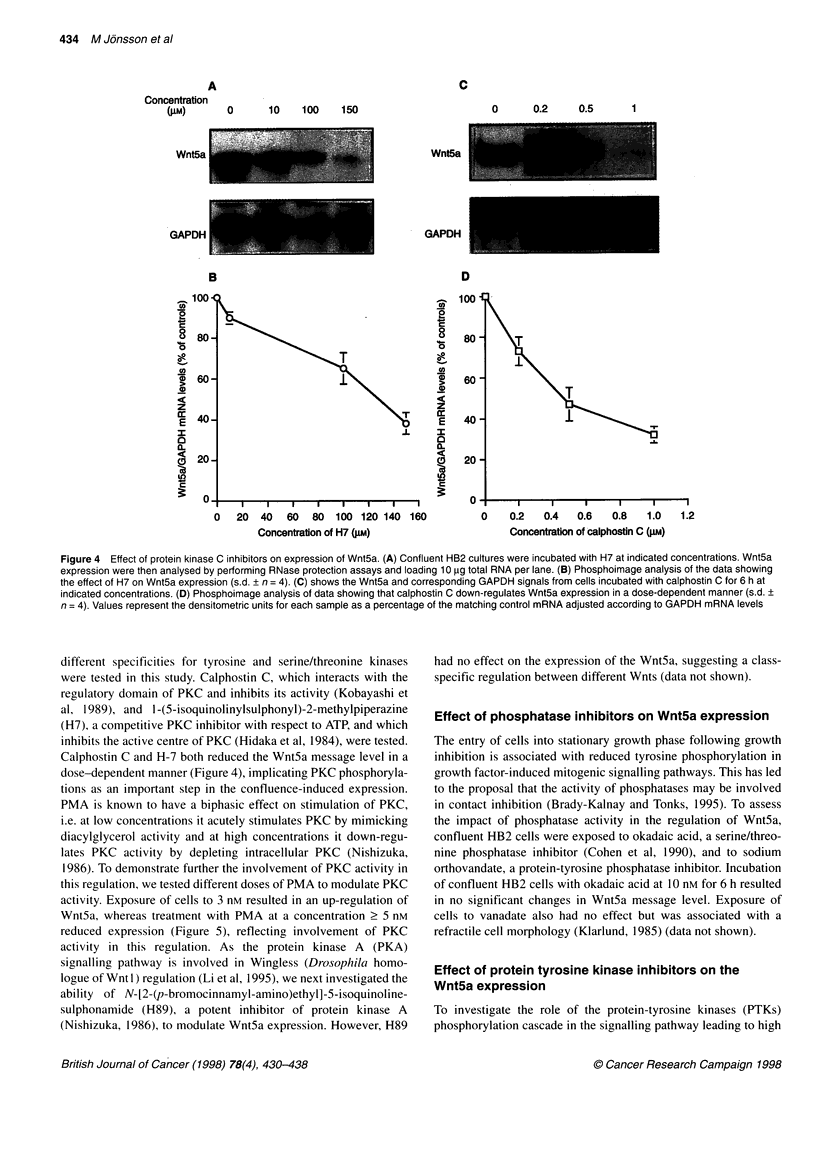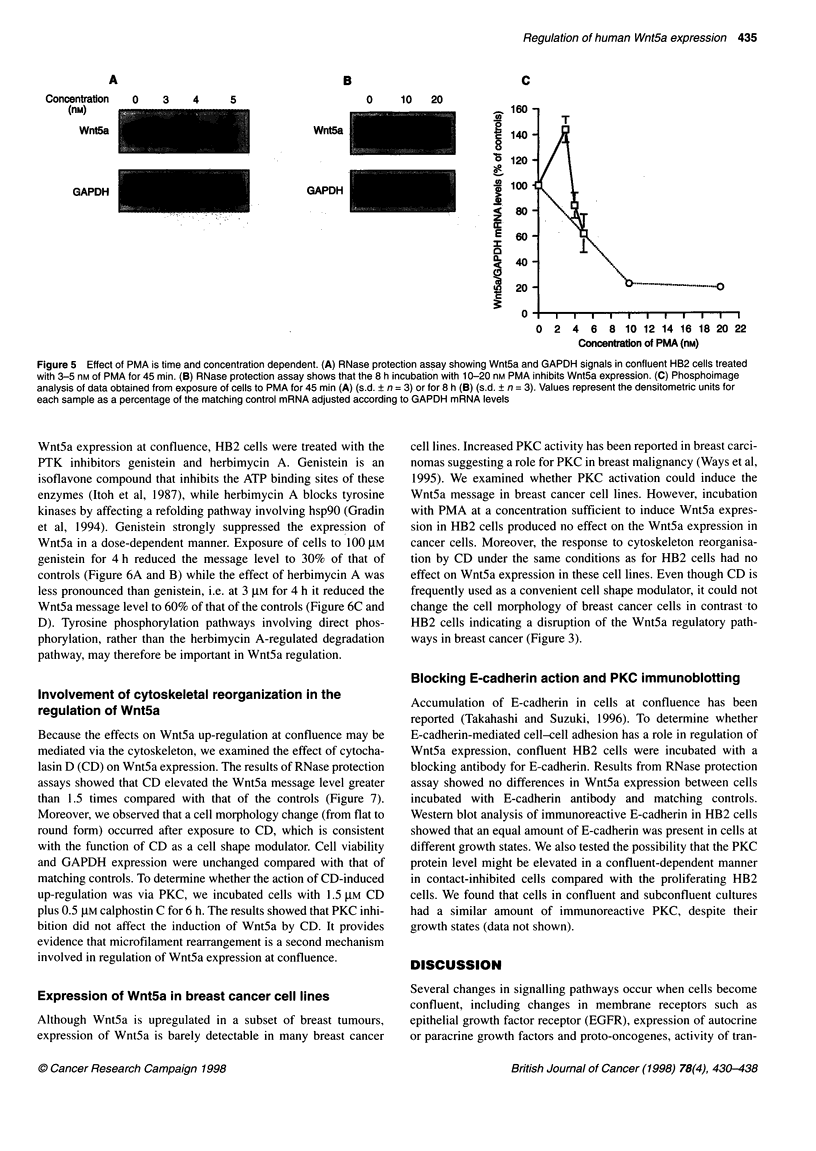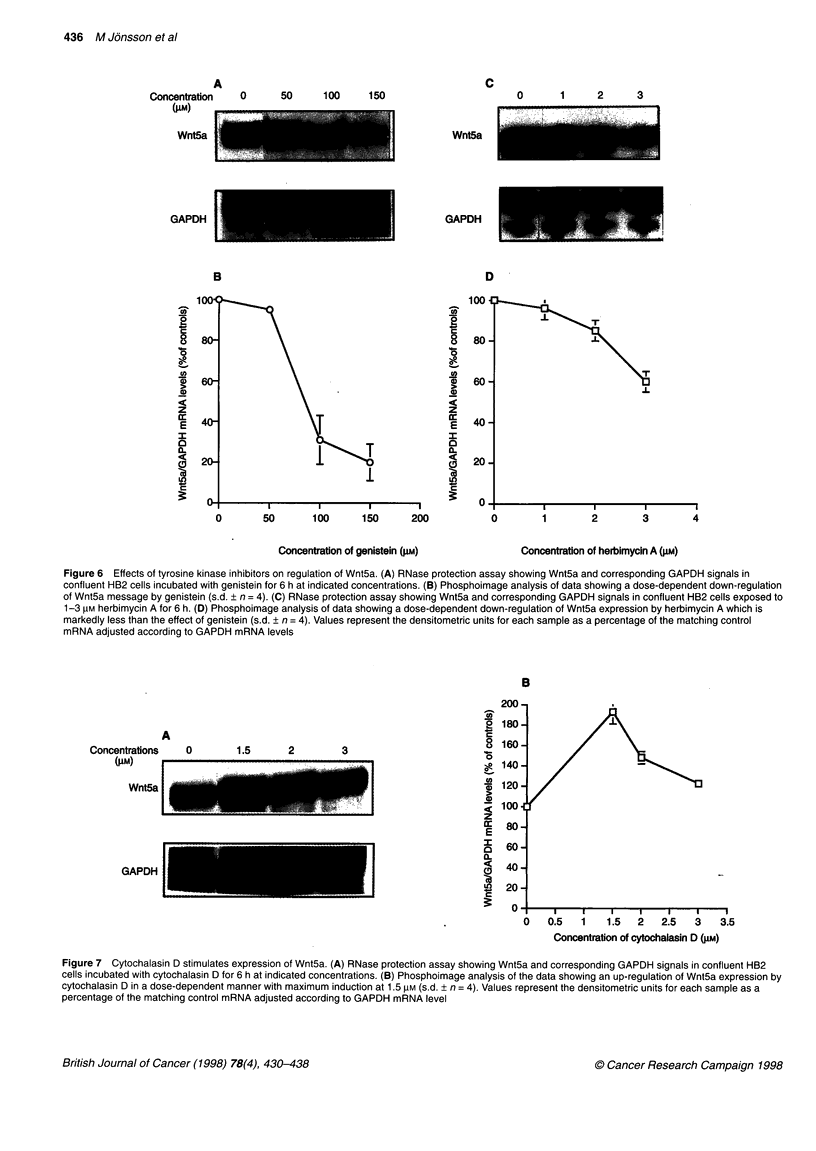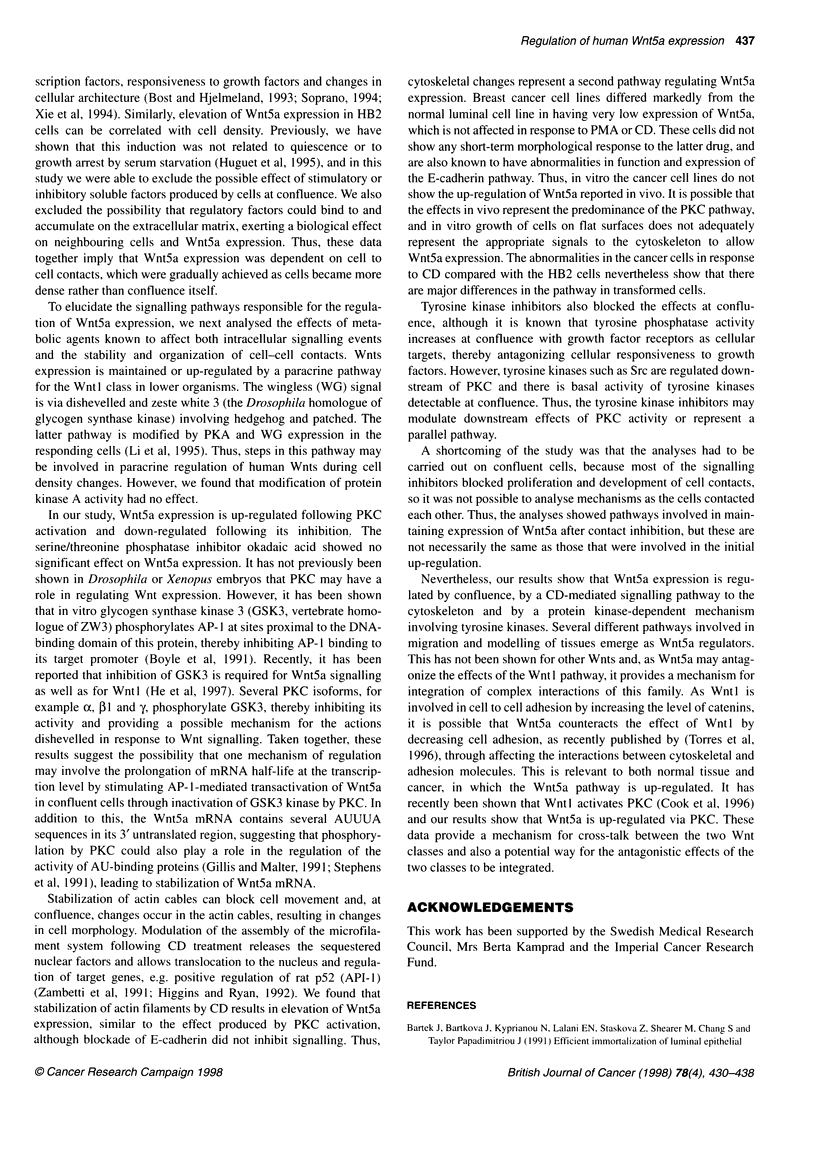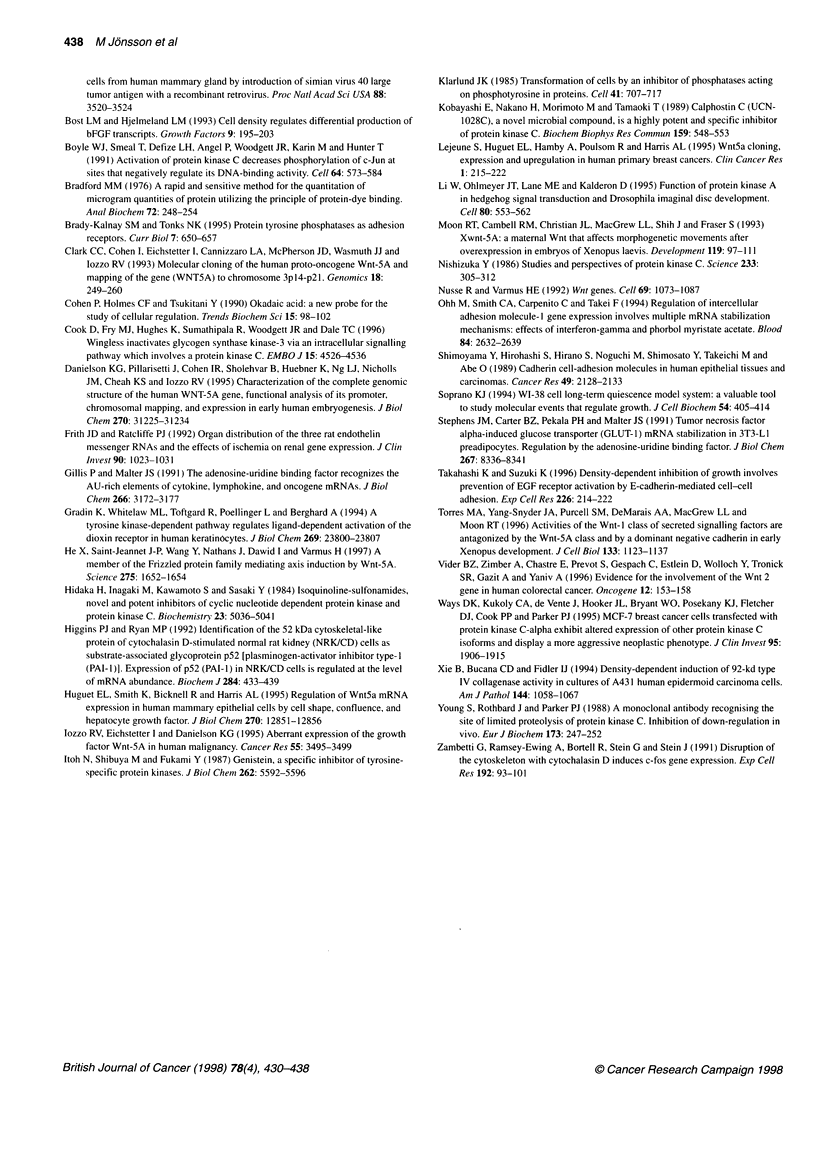# Regulation of Wnt5a expression in human mammary cells by protein kinase C activity and the cytoskeleton.

**DOI:** 10.1038/bjc.1998.511

**Published:** 1998-08

**Authors:** M. Jönsson, K. Smith, A. L. Harris

**Affiliations:** Department of Oncology, University of Lund, Sweden.

## Abstract

**Images:**


British Journal of Cancer (1998) 78(4), 430–438
© 1998 Cancer Research Campaign

### Regulation of Wnt5a expression in human mammary cells by protein kinase C activity and the cytoskeleton

M Jönsson[1], K Smith[2] and AL Harris[2]

[1]Department of Oncology, University of Lund, S-221 85 Lund, Sweden; [2]Growth Factors Group, Imperial Cancer Research Fund, University of Oxford, Institute of Molecular Medicine, John Radcliffe Hospital, Headington, Oxford OX3 9DU, UK

**Summary** The Wnts can be classified into two classes based on their ability to transform cells. The Wnt5a class can antagonize the effects of transforming Wnts partly through effects on cell migration. To understand the mechanisms of regulation of Wnt5a, we investigated its expression in human normal and breast cancer cell lines. Elevation of Wnt5a in HB2, a normal breast epithelial cell line, was linearly correlated with cell density, but this did not occur in cancer cell lines. We examined intracellular events responsible for the regulation of Wnt5a by cell to cell contacts, using various metabolic agents known to affect signal transduction pathways. Agents that selectively blocked protein kinase C (calphostin C) or protein tyrosine kinases (genistein) reduced the level of Wnt5a expression markedly. Protein kinase C activation by phorbol 12-myristate 13-acetate up-regulated Wnt5a partly through prolongation of Wnt5a mRNA half-life. Cytoskeleton reorganization following cytochalasin D treatment caused an induction of Wnt5a, which was associated with changes in cell morphology. Calphostin C did not block these effects, showing that protein kinase C is acting upstream of cytoskeletal modulation. However, the cancer cell lines treated with cytochalasin D showed no changes in cell morphology or Wnt5a induction, suggesting disruption of this regulatory pathway in cancer.

**Keywords:** human Wnt5a; regulation; kinase C; cytoskeleton

Morphogenesis in multicellular organisms requires a variety of cell to cell signals, among which local cell–cell signalling events are of particular importance. Wnt proteins are secreted proteins and are known to be such local signalling factors (Nusse and Varmus, 1992). The extracellular Wnt signal is transmitted and sensed by other cells in an auto- or paracrine way. The coordination of Wnt signals with other internal signals to induce the appropriate changes in other gene activities is poorly understood. The diversity of function between these genes, when overexpressed in *Xenopus* embryos, allows them to be divided into two distinct classes; the Wnt1 class, which includes Wnt1, 3a, 8 and 8b and which promotes axis duplication; and the Wnt5a class, which includes Wnt5a, 4 and 11, which alter morphogenetic movements. The members of the Wnt5a class are thought to antagonize the mitogenic stimulatory effect of the Wnt1 class by decreasing calcium-dependent cell adhesion by an unknown mechanism (Torres et al, 1996).

The signalling pathway used by Wnt5a class is poorly understood. In *Xenopus* embryos, ectopic expression of Wnt5a alters the morphogenetic movement by blocking the normal elongation of blastula cap explants induced by activin, suggesting a function for Wnt5a in blocking cell motility (Moon et al, 1993). This function is not yet understood in mammalian cells, but down-regulation of Wnt5a by hepatocyte growth factor (HGF), followed by cell branching in collagen (Huguet et al, 1995) and the sequence homology between human and *Xenopus* Wnt5a (90% at amino acid level), supports a similar role for human Wnt5a in controlling cell movement. In previous studies we have shown that Wnt5a is up-regulated in benign and malignant breast tumours, ten- and fourfold respectively, compared with normal breast tissue (Lejeune et al, 1995). Moreover, it has also been documented that Wnt5a is up-regulated in several other cancer types, such as lung, prostate cancer and malignant melanoma (Iozzo et al, 1995), as well as in colon cancer (Vider et al, 1996). The mechanisms involved in up-regulation of Wnt5a in cancer have not been elucidated so far, but as several Wnts, including Wnt5a, are widely expressed in both embryos and adult tissue they may, therefore, have a role in maintenance of adult tissue organization.

To study the cellular response to Wnt5a signal and the mechanisms involved in the regulation of Wnt5a, the HB2 cell line has been shown to be a suitable cell model. We have previously shown that Wnt5a is regulated by confluence, cell shape transition and HGF in this cell line (Huguet et al, 1995). In this study, we have investigated the signalling pathways involved in the regulation of Wnt5a expression in cell density and cell to cell contact. We have found that Wnt5a expression was regulated by increasing cell density in HB2 cells, with a peak at late confluence. This may provide an additional mechanism through which Wnt5a inhibits cell motility beyond cell confluence. We have also shown that protein kinase C (PKC), tyrosine kinase activities and cytoskeleton rearrangement are involved in the regulation of Wnt5a at cell confluence. The pathways by which PKC regulates the Wnt5a message level may thus be relevant to Wnt5a up-regulation in cancer, as well as in normal development, providing a link between regulatory pathways of kinases involved in many processes in adult tissues, including carcinogenesis and the pathways of embryonic differentiation.

*Received 24 July 1997*
*Revised 4 February 1998*
*Accepted 5 February 1998*

*Correspondence to:* M Jönsson, Division of Experimental Pathology, Lund University, University Hospital MAS, Entrance 78, SE-205 02 Malmö, Sweden

### MATERIALS AND METHODS

### Cell culture

Mycoplasma-free HB2 cells [a subclone of the MTSV1–7 line (Bartek et al, 1991)] obtained from Dr Joyce Taylor-Papadimitriou

(Imperial Cancer Research Fund, Lincoln's Inn Fields, UK) were cultured as described previously (Huguet et al, 1995). Briefly, cells were seeded at different densities into a 10-cm Beckton-Dickinson tissue culture plate in Dulbecco's modified Eagle medium (DMEM) (Clare Hall Laboratories, Imperial Cancer Research Fund, UK) supplemented with 10% fetal calf serum (FCS), 10 μg $ml^{-1}$ bovine insulin and 5 μg $ml^{-1}$ hydrocortisone in 95% air, 5% carbon dioxide, at 37°C. For analysis of cell density-dependent expression of Wnt5a, HB2 cells were plated at different densities. Cells were harvested when they reached a cell density between early subconfluence (150 cells $mm^{-2}$ ≤ 5% confluence) and late confluence (1500 cells $mm^{-2}$ ≥ 95% confluence). Breast cancer cell lines MCF-7, T-47D, MDA-MB-361 and BT-474 (from the American Type Culture Collection) were cultured in DMEM supplemented with 10% FCS and incubated at the same conditions as described for HB2 cells. All laboratory reagents were from Sigma unless otherwise specified.

### Preparation of RNA from subconfluent cells cultured in various conditions

To investigate whether the differences in Wnt5a expression between cells growing in different states depended upon soluble factors, subconfluent cultures were grown in conditioned medium from confluent cell cultures for 24 h and Wnt5a expression was compared with cells grown normally. The expression of Wnt5a in cells growing to subconfluence on a matrix extracted from confluent HB2 cells by repeatedly freezing and thawing the cells was also examined. To exclude the effect of supplements on Wnt5a expression, HB2 cells were grown in medium with and without each supplement. Total RNA was prepared from these cultures and analysed by ribonuclease (RNase) protection assays.

### RNase protection assay

Antisense [α-$^{32}$P]CTP (Amersham) transcripts of Wnt5a were generated from a 384-basepair fragment of the gene cloned in the plasmid bluescript KS+ (Stratagene). Antisense transcripts were generated from the construct using T7 RNA polymerase after linearization with *Eco*RV. For the glyceraldehyde-3-phosphate dehydrogenase (GAPDH) probe, which was used as internal control, a 120-basepair fragment of the GAPDH was cloned in the plasmid bluescript KS+ to generate antisense GAPDH transcripts as described previously (Huguet et al, 1995). RNase protection assays were performed on 10 μg of total RNA as described previously. The RNA was analysed on a 1% agarose gel to check purity and quantified by spectrophotometry at 260 nm. Total RNA was hybridized overnight at 55°C with labelled antisense probes. Exposing cells to different drugs may modulate GAPDH expression, thereby questioning the validity of GAPDH as loading control. To circumvent this problem, we spiked all samples with 1 μg of total RNA from K562 cells (which strongly overexpress α-1-globin) and included a [α-$^{32}$P]CTP labelled antisense α-globin mRNA to the hybridization solutions. This hybridizes to the α-globin mRNA in the spike (Frith and Ratcliffe, 1992), thereby providing an external loading control.

### Image analysis

Autoradiographs of RNase protection assay were scanned using a phosphoimager to quantify the intensity of Wnt5a, GAPDH and globin spike signals. Wnt5a mRNA value for each sample was normalized to GAPDH and to globin mRNA spike. The mean values of Wnt5a signal intensities were compared with that of matched controls in each assay and are represented as a percentage of the value for Wnt5a mRNA in control samples. All values represent a minimum of three different measurements in at least three independent experiments.

### Measuring half-life of Wnt5a mRNA

Wnt5a mRNA half-life was measured using actinomycin D (Act D). Act D was solubilized in dimethylsulphoxide (DMSO) as a stock solution of 10 mg $ml^{-1}$ and used at a final concentration of 10 μg $ml^{-1}$. The drug was added to confluent HB2 cultures and incubated in duplicate plates with matching controls. Wnt5a mRNA was then analysed at 0.5–4 h after addition of Act D. To investigate the effect of activation of PKC on Wnt5a mRNA half-life, confluent cells were preincubated in the presence and absence of 3 nM phorbol 12-myristate 13-acetate (PMA) for 45 min followed by washing three times with phosphate-buffered saline (PBS). Act D (10 μg $ml^{-1}$) was subsequently added and the cells were incubated for 2–4 h.

### Use of metabolic agents

The metabolic agents used in this study as modulators of intracellular signalling events are listed in Table 1. In all assays, HB2 cells were grown to confluence to obtain the maximum induction of Wnt5a. Drugs were then applied to the culture and their possible effect on Wnt5a expression was analysed after 4–6 h incubation (a

**Table 1** List of metabolic agents used in the present study

| Metabolic agent | Effect | Solvent/stock (mM) | Concentrations studied | Average concentrations used in the other studies |
|---|---|---|---|---|
| H7 | Inhibits serine threonine kinases | DMSO/100 | 1–150 μM | 1–150 μM |
| Herbimycin A | Inhibits tyrosine kinases | DMSO/1 | 1–3 μM | 0.1–15 μM |
| Okadaic acid | Inhibits serine threonine kinases | Acetone/0.1 | 0.1–100 nM | 0.1–150 nM |
| Cytochalasin D | Inhibits actin polymerization | DMSO/10 | 1–3 μM | 0.2–10 μM |
| Calphostin C | Inhibits PKC | DMSO/100 | 0.1–1 nM | 0.1–10 nM |
| Genistein | Inhibits tyrosine kinases | DMSO/100 | 50–150 μM | 20–150 μM |
| PMA | Stimulates PKC | DMSO/10 | 1–20 nM | 1–100 nM |
| Sodium vanadate | Inhibits tyrosine phosphatase | $H_2O$/0.1 | 1–150 μM | 1–200 μM |
| H89 | Inhibits PKA | DMSO/100 | 50–100 μM | 10–150 μM |
| Actinomycin D | Inhibits RNA synthesis | DMSO/10 | 10 μM | 5–15 μM |

© Cancer Research Campaign 1998

time generally sufficient to obtain the full effect of the drugs). Dose–response studies were carried out to optimize concentrations and solvent controls were used in test plates. Agents were prepared as stocks in minimum volumes of solvents, e.g. in DMSO or acetone, to reduce the solvent concentration in assays below 0.1% (v/v). Cell viability was assessed by cell morphology using an inverted microscope or by trypan blue exclusion. All agents were generally used at a concentration ranging from 1× to 100× the inhibition constants ($K_i$), or at concentrations causing 50% inhibition ($IC_{50}$) measured in vitro. Throughout the experiments cells were incubated in serum- and supplement-free medium, which was changed 3 h before drug addition.

### Western blot analyses

Cells from confluent or subconfluent HB2 cultures were washed and sonicated in ice-cold buffer A [50 mM Tris-HCI (pH 7.4, 20°C), 150 mM sodium chloride, 2 mM EDTA, 1 mM EGTA, 1 mM sodium orthovanadate, 50 mM sodium fluoride, 1 mM sodium pyrophosphate, 2 mM PMSF, 1 μg ml$^{-1}$ aprotinin, 1 μg ml$^{-1}$ leupeptin and 0.1% SDS]. All steps were carried out on ice or at 4°C. Protein concentration was determined by the method of Bradford using BSA as a standard (Bradford, 1976). A 50-μg aliquot of total protein from each sample was separated by 8% SDS-polyacrylamide gel electrophoresis under reducing conditions. The proteins were transferred electrophoretically onto a polyvinylidene fluoride membrane (Immobilon P, Milipore). The membrane was then probed with the anti-PKC monoclonal antibody, MC5 (Young et al, 1988). The antigen–antibody complex was detected using peroxidase-conjugated rabbit anti-mouse IgG, followed by use of an enhanced chemiluminescence (ECL) detection system (Amersham). Mouse brain extract was used as positive control.

To study the effect of E-cadherin extracellular domain blocking on Wnt5a expression, confluent cells were incubated with 50 mg ml$^{-1}$ HECD-1, mouse anti E-cadherin monoclonal antibody (Shimoyama et al, 1989) or isotype-matched control mouse IgG for 16 h. Total RNA was extracted from cells and used for RNase protection assay. The level of E-cadherin protein in confluent and subconfluent HB2 cells was measured using immunoblotting as described above.

### RESULTS

### Effect of cell density on the expression of Wnt5a in HB2 cells

To' demonstrate cell density-dependent expression of Wnt5a, single-cell suspensions were plated at a range of different cell densities. Cells were harvested and counted when the plate with the highest cell number reached confluence. Results from RNase protection assays showed a linear correlation between increased cell density and Wnt5a message level, i.e. lowest expression at early subconfluence compared with the highest message level in confluent cells (Figure 1). To clarify whether the elevation of Wnt5a by cell density was due to a gradual accumulation of stimulatory or inhibitory factors in conditioned medium, HB2 cells were grown to subconfluence in conditioned medium from confluent cells. However, Wnt5a expression in this subconfluent culture was similar to that of matched controls. As Wnt5a is secreted from cells to the extracellular matrix, we next examined whether

**Figure 1** Regulation of Wnt5a expression by cell density. (**A**) RNase protection assay showing Wnt5a and corresponding GAPDH signals in HB2 cells grown to different densities. Cells were harvested by scraping cells from the plates at various cell densities with the lowest density corresponding to 150 cells mm$^{-2}$ and highest density at 1500 cells mm$^{-2}$. Ten μg of total RNA was used for each sample in RNAase protection assay. Wnt5a mRNA levels present densitometer units adjusted according to GAPDH mRNA levels. (**B**) Phosphoimage analysis of the data showing a linear correlation between up-regulation of Wnt5a and increased cell density (s.d. ± $n$ = 3)

growing subconfluent cells on a confluent cell matrix could increase the level of Wnt5a expression. We found that cells grown on a matrix prepared from confluent HB2 cells had a similar level of expression to that of controls (data not shown). Thus, these results indicate that expression of Wnt5a is independent of soluble factors or other proteins present on the extracellular matrix, but is regulated by gradually increased cell to cell contacts.

### Half-life of Wnt5a mRNA

In order to investigate the mechanism of action of signal transduction processes on the regulation of Wnt5a expression in confluent HB2 cultures, we measured the half-life of the Wnt5a mRNA in confluent cells by blocking new mRNA synthesis with Act D. The mean half-life of Wnt5a transcripts in confluent HB2 cells was

 © Cancer Research Campaign 1998

**Figure 2** Preincubation of cells with PMA prolongs the half-life of Wnt5a mRNA (**A**) Wnt5a and corresponding GAPDH signals in confluent HB2 cells incubated in the presence or absence of Act D to determine Wnt5a mRNA half-life. (**B**) RNase protection assay showing Wnt5a and GAPDH signals in confluent HB2 cells preincubated in the presence (+) or absence (–) of 3 nM PMA for 45 min. Cells were washed three times with PBS and then continuously incubated with Act D (10 μg ml⁻¹) for indicated time intervals. (**C**) Phosphoimage analysis of the data obtained from incubation of cells with Act D or matching controls (□, s.d. ± *n*=3), or from preincubation of cells with PMA before adding Act D with matching controls. Wnt5a half-life is prolonged greater than 1.5 times in cells stimulated with PMA (○, s.d. ± *n* = 3). Wnt5a mRNA levels present densitometer units of each sample as a percentage of the matching control mRNA adjusted according to GAPDH mRNA levels

**Figure 3** Treatment of breast cancer cells by PMA and cytochalasin D has no effect on expression of Wnt5a. RNase protection assay showing Wnt5a and GAPDH signals in confluent breast cancer cells treated with 3 nM PMA for 45 min and 1.5 μM cytochalain D for 6 h. Treatment of confluent HB2 cells with the indicated concentration of PMA and CD induced the highest expression level of Wnt5a in confluent HB2 cells (see Figures 5 and 7). Lane 1 showing expression of Wnt5a in untreated HB2 which was used as a positive control for the assay. The '0' indicates expression of Wnt5a in breast cancer cell lines at 0 time estimated as 140 ± 5 min (Figure 2A). Comparison of the GAPDH signal between samples exposed to the optimized concentration of drugs and that of controls showed no significant change in GAPDH expression, proving GAPDH to be a reliable internal control.

### Regulation of Wnt5a gene transcription by PMA

Two motifs, TATT and ATTA, repeated 32 and ten times respectively, are identified at the 3′ untranslated region (UTR) of the Wnt5a sequence, implying a possible rapid degradation of Wnt5a mRNA (Clark et al, 1993). As a phorbol ester-responsive element, AP-1, is present in the Wnt5a promoter region (Danielson et al, 1995), alteration of this pathway by PKC activation could be one possible mechanism of regulation on contact. To examine whether Wnt5a mRNAs half-life was affected by treatment of cells with PMA, cells were preincubated with 3 nM PMA to modulate PKC activity prior to adding Act D. The results show that the half-life of Wnt5a was prolonged greater than 1.5 times ($t_{1/2}$ = 230 ± 6 min, Figure 2B) in response to PMA. This prolongation could reflect a corresponding increase in mRNA at the transcriptional level or stabilization of Wnt5a message by PMA, as has been reported for other labile mRNA (Ohh et al, 1994).

### Involvement of PKC in the regulation of Wnt5a

To gain insight into the intracellular mechanisms involved in confluence-induced accumulation of Wnt5a, several drugs with

**Figure 4** Effect of protein kinase C inhibitors on expression of Wnt5a. (**A**) Confluent HB2 cultures were incubated with H7 at indicated concentrations. Wnt5a expression were then analysed by performing RNase protection assays and loading 10 μg total RNA per lane. (**B**) Phosphoimage analysis of the data showing the effect of H7 on Wnt5a expression (s.d. ± *n* = 4). (**C**) shows the Wnt5a and corresponding GAPDH signals from cells incubated with calphostin C for 6 h at indicated concentrations. (**D**) Phosphoimage analysis of data showing that calphostin C down-regulates Wnt5a expression in a dose-dependent manner (s.d. ± *n* = 4). Values represent the densitometric units for each sample as a percentage of the matching control mRNA adjusted according to GAPDH mRNA levels different specificities for tyrosine and serine/threonine kinases were tested in this study. Calphostin C, which interacts with the regulatory domain of PKC and inhibits its activity (Kobayashi et al, 1989), and 1-(5-isoquinolinylsulphonyl)-2-methylpiperazine (H7), a competitive PKC inhibitor with respect to ATP, and which inhibits the active centre of PKC (Hidaka et al, 1984), were tested. Calphostin C and H-7 both reduced the Wnt5a message level in a dose–dependent manner (Figure 4), implicating PKC phosphorylations as an important step in the confluence-induced expression. PMA is known to have a biphasic effect on stimulation of PKC, i.e. at low concentrations it acutely stimulates PKC by mimicking diacylglycerol activity and at high concentrations it down-regulates PKC activity by depleting intracellular PKC (Nishizuka, 1986). To demonstrate further the involvement of PKC activity in this regulation, we tested different doses of PMA to modulate PKC activity. Exposure of cells to 3 nM resulted in an up-regulation of Wnt5a, whereas treatment with PMA at a concentration ≥ 5 nM reduced expression (Figure 5), reflecting involvement of PKC activity in this regulation. As the protein kinase A (PKA) signalling pathway is involved in Wingless (*Drosophila* homologue of Wnt1) regulation (Li et al, 1995), we next investigated the ability of *N*-[2-(*p*-bromocinnamyl-amino)ethyl]-5-isoquinoline-sulphonamide (H89), a potent inhibitor of protein kinase A (Nishizuka, 1986), to modulate Wnt5a expression. However, H89 had no effect on the expression of the Wnt5a, suggesting a class-specific regulation between different Wnts (data not shown).

### Effect of phosphatase inhibitors on Wnt5a expression

The entry of cells into stationary growth phase following growth inhibition is associated with reduced tyrosine phosphorylation in growth factor-induced mitogenic signalling pathways. This has led to the proposal that the activity of phosphatases may be involved in contact inhibition (Brady-Kalnay and Tonks, 1995). To assess the impact of phosphatase activity in the regulation of Wnt5a, confluent HB2 cells were exposed to okadaic acid, a serine/threonine phosphatase inhibitor (Cohen et al, 1990), and to sodium orthovandate, a protein-tyrosine phosphatase inhibitor. Incubation of confluent HB2 cells with okadaic acid at 10 nM for 6 h resulted in no significant changes in Wnt5a message level. Exposure of cells to vanadate also had no effect but was associated with a refractile cell morphology (Klarlund, 1985) (data not shown).

### Effect of protein tyrosine kinase inhibitors on the Wnt5a expression

To investigate the role of the protein-tyrosine kinases (PTKs) phosphorylation cascade in the signalling pathway leading to high

 © Cancer Research Campaign 1998

**Figure 5** Effect of PMA is time and concentration dependent. (**A**) RNase protection assay showing Wnt5a and GAPDH signals in confluent HB2 cells treated with 3–5 nM of PMA for 45 min. (**B**) RNase protection assay shows that the 8 h incubation with 10–20 nM PMA inhibits Wnt5a expression. (**C**) Phosphoimage analysis of data obtained from exposure of cells to PMA for 45 min (**A**) (s.d. ± $n$ = 3) or for 8 h (**B**) (s.d. ± $n$ = 3). Values represent the densitometric units for each sample as a percentage of the matching control mRNA adjusted according to GAPDH mRNA levels

Wnt5a expression at confluence, HB2 cells were treated with the PTK inhibitors genistein and herbimycin A. Genistein is an isoflavone compound that inhibits the ATP binding sites of these enzymes (Itoh et al, 1987), while herbimycin A blocks tyrosine kinases by affecting a refolding pathway involving hsp90 (Gradin et al, 1994). Genistein strongly suppressed the expression of Wnt5a in a dose-dependent manner. Exposure of cells to 100 μM genistein for 4 h reduced the message level to 30% of that of controls (Figure 6A and B) while the effect of herbimycin A was less pronounced than genistein, i.e. at 3 μM for 4 h it reduced the Wnt5a message level to 60% of that of the controls (Figure 6C and D). Tyrosine phosphorylation pathways involving direct phosphorylation, rather than the herbimycin A-regulated degradation pathway, may therefore be important in Wnt5a regulation.

### Involvement of cytoskeletal reorganization in the regulation of Wnt5a

Because the effects on Wnt5a up-regulation at confluence may be mediated via the cytoskeleton, we examined the effect of cytochalasin D (CD) on Wnt5a expression. The results of RNase protection assays showed that CD elevated the Wnt5a message level greater than 1.5 times compared with that of the controls (Figure 7). Moreover, we observed that a cell morphology change (from flat to round form) occurred after exposure to CD, which is consistent with the function of CD as a cell shape modulator. Cell viability and GAPDH expression were unchanged compared with that of matching controls. To determine whether the action of CD-induced up-regulation was via PKC, we incubated cells with 1.5 μM CD plus 0.5 μM calphostin C for 6 h. The results showed that PKC inhibition did not affect the induction of Wnt5a by CD. It provides evidence that microfilament rearrangement is a second mechanism involved in regulation of Wnt5a expression at confluence.

### Expression of Wnt5a in breast cancer cell lines

Although Wnt5a is upregulated in a subset of breast tumours, expression of Wnt5a is barely detectable in many breast cancer cell lines. Increased PKC activity has been reported in breast carcinomas suggesting a role for PKC in breast malignancy (Ways et al, 1995). We examined whether PKC activation could induce the Wnt5a message in breast cancer cell lines. However, incubation with PMA at a concentration sufficient to induce Wnt5a expression in HB2 cells produced no effect on the Wnt5a expression in cancer cells. Moreover, the response to cytoskeleton reorganisation by CD under the same conditions as for HB2 cells had no effect on Wnt5a expression in these cell lines. Even though CD is frequently used as a convenient cell shape modulator, it could not change the cell morphology of breast cancer cells in contrast to HB2 cells indicating a disruption of the Wnt5a regulatory pathways in breast cancer (Figure 3).

### Blocking E-cadherin action and PKC immunoblotting

Accumulation of E-cadherin in cells at confluence has been reported (Takahashi and Suzuki, 1996). To determine whether E-cadherin-mediated cell–cell adhesion has a role in regulation of Wnt5a expression, confluent HB2 cells were incubated with a blocking antibody for E-cadherin. Results from RNase protection assay showed no differences in Wnt5a expression between cells incubated with E-cadherin antibody and matching controls. Western blot analysis of immunoreactive E-cadherin in HB2 cells showed that an equal amount of E-cadherin was present in cells at different growth states. We also tested the possibility that the PKC protein level might be elevated in a confluent-dependent manner in contact-inhibited cells compared with the proliferating HB2 cells. We found that cells in confluent and subconfluent cultures had a similar amount of immunoreactive PKC, despite their growth states (data not shown).

### DISCUSSION

Several changes in signalling pathways occur when cells become confluent, including changes in membrane receptors such as epithelial growth factor receptor (EGFR), expression of autocrine or paracrine growth factors and proto-oncogenes, activity of tran-

© Cancer Research Campaign 1998 

**Figure 6** Effects of tyrosine kinase inhibitors on regulation of Wnt5a. (**A**) RNase protection assay showing Wnt5a and corresponding GAPDH signals in confluent HB2 cells incubated with genistein for 6 h at indicated concentrations. (**B**) Phosphoimage analysis of data showing a dose-dependent down-regulation of Wnt5a message by genistein (s.d. ± $n$ = 4). (**C**) RNase protection assay showing Wnt5a and corresponding GAPDH signals in confluent HB2 cells exposed to 1–3 μM herbimycin A for 6 h. (**D**) Phosphoimage analysis of data showing a dose-dependent down-regulation of Wnt5a expression by herbimycin A which is markedly less than the effect of genistein (s.d. ± $n$ = 4). Values represent the densitometric units for each sample as a percentage of the matching control mRNA adjusted according to GAPDH mRNA levels

**Figure 7** Cytochalasin D stimulates expression of Wnt5a. (**A**) RNase protection assay showing Wnt5a and corresponding GAPDH signals in confluent HB2 cells incubated with cytochalasin D for 6 h at indicated concentrations. (**B**) Phosphoimage analysis of the data showing an up-regulation of Wnt5a expression by cytochalasin D in a dose-dependent manner with maximum induction at 1.5 μM (s.d. ± $n$ = 4). Values represent the densitometric units for each sample as a percentage of the matching control mRNA adjusted according to GAPDH mRNA level

© Cancer Research Campaign 1998

scription factors, responsiveness to growth factors and changes in cellular architecture (Bost and Hjelmeland, 1993; Soprano, 1994; Xie et al, 1994). Similarly, elevation of Wnt5a expression in HB2 cells can be correlated with cell density. Previously, we have shown that this induction was not related to quiescence or to growth arrest by serum starvation (Huguet et al, 1995), and in this study we were able to exclude the possible effect of stimulatory or inhibitory soluble factors produced by cells at confluence. We also excluded the possibility that regulatory factors could bind to and accumulate on the extracellular matrix, exerting a biological effect on neighbouring cells and Wnt5a expression. Thus, these data together imply that Wnt5a expression was dependent on cell to cell contacts, which were gradually achieved as cells became more dense rather than confluence itself.

To elucidate the signalling pathways responsible for the regulation of Wnt5a expression, we next analysed the effects of metabolic agents known to affect both intracellular signalling events and the stability and organization of cell–cell contacts. Wnts expression is maintained or up-regulated by a paracrine pathway for the Wnt1 class in lower organisms. The wingless (WG) signal is via dishevelled and zeste white 3 (the *Drosophila* homologue of glycogen synthase kinase) involving hedgehog and patched. The latter pathway is modified by PKA and WG expression in the responding cells (Li et al, 1995). Thus, steps in this pathway may be involved in paracrine regulation of human Wnts during cell density changes. However, we found that modification of protein kinase A activity had no effect.

In our study, Wnt5a expression is up-regulated following PKC activation and down-regulated following its inhibition. The serine/threonine phosphatase inhibitor okadaic acid showed no significant effect on Wnt5a expression. It has not previously been shown in *Drosophila* or *Xenopus* embryos that PKC may have a role in regulating Wnt expression. However, it has been shown that in vitro glycogen synthase kinase 3 (GSK3, vertebrate homologue of ZW3) phosphorylates AP-1 at sites proximal to the DNA-binding domain of this protein, thereby inhibiting AP-1 binding to its target promoter (Boyle et al, 1991). Recently, it has been reported that inhibition of GSK3 is required for Wnt5a signalling as well as for Wnt1 (He et al, 1997). Several PKC isoforms, for example $\alpha$, $\beta1$ and $\gamma$, phosphorylate GSK3, thereby inhibiting its activity and providing a possible mechanism for the actions dishevelled in response to Wnt signalling. Taken together, these results suggest the possibility that one mechanism of regulation may involve the prolongation of mRNA half-life at the transcription level by stimulating AP-1-mediated transactivation of Wnt5a in confluent cells through inactivation of GSK3 kinase by PKC. In addition to this, the Wnt5a mRNA contains several AUUUA sequences in its 3′ untranslated region, suggesting that phosphorylation by PKC could also play a role in the regulation of the activity of AU-binding proteins (Gillis and Malter, 1991; Stephens et al, 1991), leading to stabilization of Wnt5a mRNA.

Stabilization of actin cables can block cell movement and, at confluence, changes occur in the actin cables, resulting in changes in cell morphology. Modulation of the assembly of the microfilament system following CD treatment releases the sequestered nuclear factors and allows translocation to the nucleus and regulation of target genes, e.g. positive regulation of rat p52 (API-1) (Zambetti et al, 1991; Higgins and Ryan, 1992). We found that stabilization of actin filaments by CD results in elevation of Wnt5a expression, similar to the effect produced by PKC activation, although blockade of E-cadherin did not inhibit signalling. Thus, cytoskeletal changes represent a second pathway regulating Wnt5a expression. Breast cancer cell lines differed markedly from the normal luminal cell line in having very low expression of Wnt5a, which is not affected in response to PMA or CD. These cells did not show any short-term morphological response to the latter drug, and are also known to have abnormalities in function and expression of the E-cadherin pathway. Thus, in vitro the cancer cell lines do not show the up-regulation of Wnt5a reported in vivo. It is possible that the effects in vivo represent the predominance of the PKC pathway, and in vitro growth of cells on flat surfaces does not adequately represent the appropriate signals to the cytoskeleton to allow Wnt5a expression. The abnormalities in the cancer cells in response to CD compared with the HB2 cells nevertheless show that there are major differences in the pathway in transformed cells.

Tyrosine kinase inhibitors also blocked the effects at confluence, although it is known that tyrosine phosphatase activity increases at confluence with growth factor receptors as cellular targets, thereby antagonizing cellular responsiveness to growth factors. However, tyrosine kinases such as Src are regulated downstream of PKC and there is basal activity of tyrosine kinases detectable at confluence. Thus, the tyrosine kinase inhibitors may modulate downstream effects of PKC activity or represent a parallel pathway.

A shortcoming of the study was that the analyses had to be carried out on confluent cells, because most of the signalling inhibitors blocked proliferation and development of cell contacts, so it was not possible to analyse mechanisms as the cells contacted each other. Thus, the analyses showed pathways involved in maintaining expression of Wnt5a after contact inhibition, but these are not necessarily the same as those that were involved in the initial up-regulation.

Nevertheless, our results show that Wnt5a expression is regulated by confluence, by a CD-mediated signalling pathway to the cytoskeleton and by a protein kinase-dependent mechanism involving tyrosine kinases. Several different pathways involved in migration and modelling of tissues emerge as Wnt5a regulators. This has not been shown for other Wnts and, as Wnt5a may antagonize the effects of the Wnt1 pathway, it provides a mechanism for integration of complex interactions of this family. As Wnt1 is involved in cell to cell adhesion by increasing the level of catenins, it is possible that Wnt5a counteracts the effect of Wnt1 by decreasing cell adhesion, as recently published by (Torres et al, 1996), through affecting the interactions between cytoskeletal and adhesion molecules. This is relevant to both normal tissue and cancer, in which the Wnt5a pathway is up-regulated. It has recently been shown that Wnt1 activates PKC (Cook et al, 1996) and our results show that Wnt5a is up-regulated via PKC. These data provide a mechanism for cross-talk between the two Wnt classes and also a potential way for the antagonistic effects of the two classes to be integrated.

### ACKNOWLEDGEMENTS

This work has been supported by the Swedish Medical Research Council, Mrs Berta Kamprad and the Imperial Cancer Research Fund.

### REFERENCES

Bartek J, Bartkova J, Kyprianou N, Lalani EN, Staskova Z, Shearer M, Chang S and Taylor Papadimitriou J (1991) Efficient immortalization of luminal epithelial

© Cancer Research Campaign 1998

cells from human mammary gland by introduction of simian virus 40 large tumor antigen with a recombinant retrovirus. *Proc Natl Acad Sci USA* **88**: 3520–3524

Bost LM and Hjelmeland LM (1993) Cell density regulates differential production of bFGF transcripts. *Growth Factors* **9**: 195–203

Boyle WJ, Smeal T, Defize LH, Angel P, Woodgett JR, Karin M and Hunter T (1991) Activation of protein kinase C decreases phosphorylation of c-Jun at sites that negatively regulate its DNA-binding activity. *Cell* **64**: 573–584

Bradford MM (1976) A rapid and sensitive method for the quantitation of microgram quantities of protein utilizing the principle of protein-dye binding. *Anal Biochem* **72**: 248–254

Brady-Kalnay SM and Tonks NK (1995) Protein tyrosine phosphatases as adhesion receptors. *Curr Biol* **7**: 650–657

Clark CC, Cohen I, Eichstetter I, Cannizzaro LA, McPherson JD, Wasmuth JJ and Iozzo RV (1993) Molecular cloning of the human proto-oncogene Wnt-5A and mapping of the gene (WNT5A) to chromosome 3p14-p21. *Genomics* **18**: 249–260

Cohen P, Holmes CF and Tsukitani Y (1990) Okadaic acid: a new probe for the study of cellular regulation. *Trends Biochem Sci* **15**: 98–102

Cook D, Fry MJ, Hughes K, Sumathipala R, Woodgett JR and Dale TC (1996) Wingless inactivates glycogen synthase kinase-3 via an intracellular signalling pathway which involves a protein kinase C. *EMBO J* **15**: 4526–4536

Danielson KG, Pillarisetti J, Cohen IR, Sholehvar B, Huebner K, Ng LJ, Nicholls JM, Cheah KS and Iozzo RV (1995) Characterization of the complete genomic structure of the human WNT-5A gene, functional analysis of its promoter, chromosomal mapping, and expression in early human embryogenesis. *J Biol Chem* **270**: 31225–31234

Frith JD and Ratcliffe PJ (1992) Organ distribution of the three rat endothelin messenger RNAs and the effects of ischemia on renal gene expression. *J Clin Invest* **90**: 1023–1031

Gillis P and Malter JS (1991) The adenosine-uridine binding factor recognizes the AU-rich elements of cytokine, lymphokine, and oncogene mRNAs. *J Biol Chem* **266**: 3172–3177

Gradin K, Whitelaw ML, Toftgard R, Poellinger L and Berghard A (1994) A tyrosine kinase-dependent pathway regulates ligand-dependent activation of the dioxin receptor in human keratinocytes. *J Biol Chem* **269**: 23800–23807

He X, Saint-Jeannet J-P, Wang Y, Nathans J, Dawid I and Varmus H (1997) A member of the Frizzled protein family mediating axis induction by Wnt-5A. *Science* **275**: 1652–1654

Hidaka H, Inagaki M, Kawamoto S and Sasaki Y (1984) Isoquinoline-sulfonamides, novel and potent inhibitors of cyclic nucleotide dependent protein kinase and protein kinase C. *Biochemistry* **23**: 5036–5041

Higgins PJ and Ryan MP (1992) Identification of the 52 kDa cytoskeletal-like protein of cytochalasin D-stimulated normal rat kidney (NRK/CD) cells as substrate-associated glycoprotein p52 [plasminogen-activator inhibitor type-1 (PAI-1)]. Expression of p52 (PAI-1) in NRK/CD cells is regulated at the level of mRNA abundance. *Biochem J* **284**: 433–439

Huguet EL, Smith K, Bicknell R and Harris AL (1995) Regulation of Wnt5a mRNA expression in human mammary epithelial cells by cell shape, confluence, and hepatocyte growth factor. *J Biol Chem* **270**: 12851–12856

Iozzo RV, Eichstetter I and Danielson KG (1995) Aberrant expression of the growth factor Wnt-5A in human malignancy. *Cancer Res* **55**: 3495–3499

Itoh N, Shibuya M and Fukami Y (1987) Genistein, a specific inhibitor of tyrosine-specific protein kinases. *J Biol Chem* **262**: 5592–5596

Klarlund JK (1985) Transformation of cells by an inhibitor of phosphatases acting on phosphotyrosine in proteins. *Cell* **41**: 707–717

Kobayashi E, Nakano H, Morimoto M and Tamaoki T (1989) Calphostin C (UCN-1028C), a novel microbial compound, is a highly potent and specific inhibitor of protein kinase C. *Biochem Biophys Res Commun* **159**: 548–553

Lejeune S, Huguet EL, Hamby A, Poulsom R and Harris AL (1995) Wnt5a cloning, expression and upregulation in human primary breast cancers. *Clin Cancer Res* **1**: 215–222

Li W, Ohlmeyer JT, Lane ME and Kalderon D (1995) Function of protein kinase A in hedgehog signal transduction and Drosophila imaginal disc development. *Cell* **80**: 553–562

Moon RT, Cambell RM, Christian JL, MacGrew LL, Shih J and Fraser S (1993) Xwnt-5A: a maternal Wnt that affects morphogenetic movements after overexpression in embryos of Xenopus laevis. *Development* **119**: 97–111

Nishizuka Y (1986) Studies and perspectives of protein kinase C. *Science* **233**: 305–312

Nusse R and Varmus HE (1992) *Wnt* genes. *Cell* **69**: 1073–1087

Ohh M, Smith CA, Carpenito C and Takei F (1994) Regulation of intercellular adhesion molecule-1 gene expression involves multiple mRNA stabilization mechanisms: effects of interferon-gamma and phorbol myristate acetate. *Blood* **84**: 2632–2639

Shimoyama Y, Hirohashi S, Hirano S, Noguchi M, Shimosato Y, Takeichi M and Abe O (1989) Cadherin cell-adhesion molecules in human epithelial tissues and carcinomas. *Cancer Res* **49**: 2128–2133

Soprano KJ (1994) WI-38 cell long-term quiescence model system: a valuable tool to study molecular events that regulate growth. *J Cell Biochem* **54**: 405–414

Stephens JM, Carter BZ, Pekala PH and Malter JS (1991) Tumor necrosis factor alpha-induced glucose transporter (GLUT-1) mRNA stabilization in 3T3-L1 preadipocytes. Regulation by the adenosine-uridine binding factor. *J Biol Chem* **267**: 8336–8341

Takahashi K and Suzuki K (1996) Density-dependent inhibition of growth involves prevention of EGF receptor activation by E-cadherin-mediated cell–cell adhesion. *Exp Cell Res* **226**: 214–222

Torres MA, Yang-Snyder JA, Purcell SM, DeMarais AA, MacGrew LL and Moon RT (1996) Activities of the Wnt-1 class of secreted signalling factors are antagonized by the Wnt-5A class and by a dominant negative cadherin in early Xenopus development. *J Cell Biol* **133**: 1123–1137

Vider BZ, Zimber A, Chastre E, Prevot S, Gespach C, Estlein D, Wolloch Y, Tronick SR, Gazit A and Yaniv A (1996) Evidence for the involvement of the Wnt 2 gene in human colorectal cancer. *Oncogene* **12**: 153–158

Ways DK, Kukoly CA, de Vente J, Hooker JL, Bryant WO, Posekany KJ, Fletcher DJ, Cook PP and Parker PJ (1995) MCF-7 breast cancer cells transfected with protein kinase C-alpha exhibit altered expression of other protein kinase C isoforms and display a more aggressive neoplastic phenotype. *J Clin Invest* **95**: 1906–1915

Xie B, Bucana CD and Fidler IJ (1994) Density-dependent induction of 92-kd type IV collagenase activity in cultures of A431 human epidermoid carcinoma cells. *Am J Pathol* **144**: 1058–1067

Young S, Rothbard J and Parker PJ (1988) A monoclonal antibody recognising the site of limited proteolysis of protein kinase C. Inhibition of down-regulation in vivo. *Eur J Biochem* **173**: 247–252

Zambetti G, Ramsey-Ewing A, Bortell R, Stein G and Stein J (1991) Disruption of the cytoskeleton with cytochalasin D induces c-fos gene expression. *Exp Cell Res* **192**: 93–101

*© Cancer Research Campaign 1998*